# Rodent Models of Glaucoma: How Mice and Rats Can Help Human Vision Move Out of the Woods and Into the Light

**DOI:** 10.3390/cells14211648

**Published:** 2025-10-22

**Authors:** Lorenza Di Marsico, Arianna Sturlese Verduri, Silvia Marracci, Rosario Amato, Massimo Dal Monte

**Affiliations:** Department of Biology, University of Pisa, 56125 Pisa, Italy; l.dimarsico@studenti.unipi.it (L.D.M.); a.verduri1@studenti.unipi.it (A.S.V.); silvia.marracci@unipi.it (S.M.)

**Keywords:** intraocular pressure, genetic models, aqueous humor dynamics, retinal ganglion cell degeneration

## Abstract

Glaucoma represents a social and economic burden due to both its increasing incidence and the lack of knowledge about its physiopathology and treatment strategies. The main factor hindering progress in glaucoma research is the disease’s heterogeneity, which depends on both genetic and environmental factors. This limitation directly affects glaucoma research, posing obstacles to the elucidation of risk factors, disease mechanisms, and treatment strategies. Therefore, the need emerges to integrate pre-clinical experimental observations from different experimental models to recapitulate different aspects of the disease and achieve a successful translation to clinics. Here, we reviewed the glaucoma models that are currently available for basic and translational research, with a specific focus on models based on rodents. Regarding genetic glaucoma models, we considered the main hallmarks and limitations of DBA/2J, glutamate/aspartate transporter/excitatory amino acid carrier 1, myocilin, connective tissue growth factor, optineurin, purinergic receptor 2Y, caveolin 1, and endothelin-1 mice. Regarding other glaucoma models, we considered rodent models based on intraocular pressure elevation via perturbation of aqueous humor dynamics or on direct degeneration of retinal ganglion cells via physical or chemical damage.

## 1. Introduction

The term glaucoma refers to a group of optic neuropathies characterized by optic nerve cupping; progressive degeneration of retinal ganglion cells (RGCs), which ultimately die through apoptosis; and progressive thinning of the nerve fiber layer (NFL). Vision loss occurs primarily in the periphery of the visual field, resulting in the so-called tunnel vision typical of glaucoma patients [1]. With a prevalence of 3.54% in the population over 40 years, glaucoma is the leading cause of irreversible blindness worldwide. The number of affected people, currently estimated at around 80 million, is expected to increase to 112 million by 2040 due to the increase in life expectancy [2]. In Europe, the total number of glaucoma patients was estimated to be 12.26 million people in 2024, and despite an expected decline in the European population of 11.8%, the number of glaucoma patients is expected to increase to 13.52 million by 2050 [3]. The primary risk factor of glaucoma is an increase in intraocular pressure (IOP). This increase is slow and progressive [4], and it derives from an imbalance between aqueous humor production at the level of the ciliary body and its drainage through the trabecular meshwork (TM) located in the iridocorneal angle. The increase in IOP is asymptomatic; therefore, there is a high risk of detecting the disease only in an advanced stage [5].

**Figure 1:**
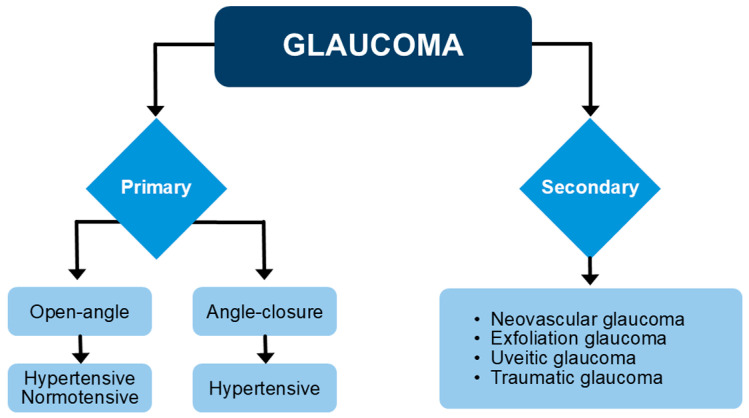
Primary and secondary types of glaucoma.

Different glaucoma subtypes can be recognized (Figure 1).

Primary glaucoma, the most common type of glaucoma, refers to glaucoma that is not caused by another condition, while secondary glaucoma is caused by another identified condition, such as, for instance, iris neovascularization, lens exfoliation, iris inflammation, or trauma [6]. Among the primary forms, primary open-angle glaucoma (POAG) affects about 90% of glaucoma patients and is related to problems in the outflow of the aqueous humor throughout the TM. This form may occur in the presence of increased IOP, but it can also appear with normal IOP, as in the case of the so-called normotensive glaucoma (NTG), which may be due to alterations of the neurovascular unit, mechanical stress, or genetic components, although the mechanisms of NTG pathogenesis still remain unclear [7]. A second type of primary glaucoma is primary angle-closure glaucoma (PACG), in which an increase in IOP derives from mechanical closure of the TM. In PACG, the IOP increase may be higher than in POAG, and based on its timing, PACG may be categorized into an acute and chronic form [8].

The first-line intervention for many glaucoma patients is the use of IOP-lowering drugs. The options for pharmacological intervention have evolved over time, and presently, they rely on α2-adrenergic agonists, β-adrenergic antagonists, cholinergic agonists, carbonic anhydrase inhibitors, prostaglandin analogs, rho kinase inhibitors, and hyperosmotic agents (Table 1). When IOP-lowering drugs fail in decreasing IOP, surgical options (laser therapy, trabeculectomy, or minimally invasive glaucoma surgeries) may be considered [9].

The molecular mechanisms underlying glaucoma pathogenesis and glaucoma progression are not completely understood. To investigate these aspects, together with the testing of possible drug targets and of putative treatments, different animal models of the disease have been developed. Mimicking glaucomatous degeneration in vivo relies on the reproduction of characteristic structural damage that defines the human condition, such as the specific loss of RGCs or axon fibers within the optic nerve, and optic cup excavation [10]. These types of damage are often, but not necessarily, dependent upon IOP elevation obtained through either genetic manipulation (or genetic background), or surgical interventions. It is important to note that no single model completely mirrors the human pathology, which is complex and multifactorial, derived from interactions between genetic and environmental factors. In addition, anatomic differences in the eye and in the optic nerve head, as well as differences in specific RGC types, also contribute to the difficulty of finding a single “perfect” animal model. However, a huge range of animal models has been developed over time, including non-human primates, dogs, cats, rabbits, birds, and zebrafish [11,12]. Among them, those developed in rodents are the most widely used; therefore, the present review focuses on the main glaucoma models developed in mice and rats. The versatility of rodent models, their relatively low cost, the similarity of the eye anterior chamber (involved in aqueous humor drainage) to that of the human eye, and the development of a rodent-specific rebound tonometer are some of the features that have made these models increasingly popular over time. However, these models also have some drawbacks, such as the presence of a glial lamina cribrosa, which is different from the fibrous lamina cribrosa in humans.

A broad classification of rodent models of glaucoma includes genetic models, characterized by spontaneous glaucoma development, and induced glaucoma models, in which glaucoma is induced through surgery or pharmacological treatments. Although similar phenotypes are scattered across different models, either genetic or induced, this classification allows us to separately consider models, investigating the impact of particular genetic traits on glaucoma development (genetic models) and models through which the damage to RGCs and optic nerve can be modulated to induce acute or chronic retinal damage (models of induced glaucoma). The aim of this review is to recapitulate the main rodent models of glaucoma, considering their strengths and weaknesses and highlighting how they may be used in a translational perspective.

## 2. Methods

The present review is purely descriptive and does not represent a systematic analysis of the available literature. The database PubMed was searched for papers published after 2010. We also screened the references from retrieved papers to identify additional related pre-clinical studies using mouse or rat models.

We searched the literature using the following terms: “glaucoma” [title/abstract] and (“mouse” [title/abstract] or “rat” [title/abstract]).

The inclusion criteria were as follows: (1) papers directly referring to RGC and/or optic nerve damage; (2) papers assessing the entity and the time course of the damage; (3) papers published in English. The exclusion criteria were as follows: (1) papers using in vitro and not in vivo models; (2) papers in which the use of rodent models had a marginal role in the context of the study; (3) papers published in languages other than English; (4) papers without abstracts; (5) papers without accessible full texts.

The researchers independently reviewed titles and abstracts based on the eligibility criteria. All disputes were resolved through discussion and debate among the authors. For research with similar findings, only the most complete publication was considered.

## 3. Genetic Models of Glaucoma

Inherited models of glaucoma generally show a higher dynamicity in glaucoma onset and progression with respect to animal models of induced glaucoma. In addition, glaucoma features are more uniform in genetic models than in surgically induced models, where disease hallmarks show a higher degree of variability. Moreover, the genetic models allow investigations of the link between particular genetic traits and the development of the disease, thus being an invaluable tool in developing therapeutic interventions. On the other hand, genetic models generally have a very slow onset of the disease, which requires long-term investigational studies. The main genetic models of glaucoma are summarized in Table 2.

### 3.1. DBA/2J

The DBA/2J mouse is a common model of experimental glaucoma. It is characterized by spontaneous development of iris pigment dispersion and stromal atrophy caused by spontaneous mutations in two melanosome-related genes: *Tyrp1* (*Tyrp1^b^*) and *Gpnmb* (*Gpnmb^R150X^*) [13,14,15]. These mice develop age-related and asynchronous IOP elevation, which typically begins at around 8–9 months of age, peaking between 10 and 12 months. This IOP increase is associated with progressive optic nerve damage, with about 34% axonal loss at 10 months and over 80% by 17–19 months [15]. Histological changes are mainly confined to the RGC layer, while retinal neurons in different layers are largely unaffected, even in older animals [16]. Although C57BL/6J mice carrying the same mutations as the DBA/2J mice develop similar iris abnormalities, they show a much lower incidence of ocular hypertension, suggesting that the genetic background influences the susceptibility to IOP elevation and glaucomatous damage [17,18].

DBA/2J mice have been widely used to study the pathophysiology of ocular hypertension-induced RGC loss. Neurodegeneration firstly affects the optic nerve, with axon damage beginning posterior to the laminar region and then retrogradely moving to the RGC soma, where it is observed about 1 month later [19]. Several pathological features are observed during disease progression, including axonal degeneration at the glial lamina, structural and functional changes in RGCs, mitochondrial and vascular dysfunction, and neuroinflammatory responses [20]. Glial cells, including microglia, Müller cells, and optic nerve head astrocytes, have been implicated in mediating neurodegeneration [21,22]. Astrocytes in the optic nerve head undergo early morphological changes before RGC loss becomes evident [23], and transcriptomic data support their involvement in disease progression [24,25,26]. Microglial activation is also correlated with RGC death, and inhibiting microglia has been shown to increase RGC survival [27,28,29]. Recent studies have revealed that Müller cell activation occurs as early as 6 months of age, preceding the elevation of IOP. This early gliosis is accompanied by upregulation of hypoxia-inducible factor-1α and the vascular endothelial growth factor, indicating a pro-inflammatory and pro-angiogenic environment that may compromise the blood–retinal barrier and contribute to RGC loss. In contrast, significant astrocyte gliosis is observed later, around 10 months of age, coinciding with the onset of IOP elevation and RGC degeneration [30]. Of note, RGC degeneration is further increased in concomitance with additional pathologies such as diabetes [31].

The DBA/2J model has been extensively used in preclinical testing of neuroprotective therapies. Agents such as 17-β-estradiol, erythropoietin, glial cell line-derived neurotrophic factor, nicotinamide, and endothelin receptor antagonists or nutraceutical approaches with functional foods rich in antioxidants have been demonstrated to exert protective effects in this model [24,27,32,33,34,35,36]. On the other hand, despite the significant contribution of this model to glaucoma research, functional analyses, such as optical coherence tomography, fundus imaging, and electroretinograms, are difficult due to poor pupil dilation in response to mydriatic drugs, and corneal calcification makes accurate IOP measurements by rebound tonometry difficult [37]. In addition, neurodegeneration and thinning of the inner retinal layers evidenced in this model are not strictly dependent on IOP elevation. Indeed, the reduction in RGC density in the DBA/2J retina is not linearly correlated with IOP levels [38], suggesting that IOP-independent mechanisms may trigger neurodegenerative processes.

### 3.2. Glutamate/Aspartate Transporter/Excitatory Amino Acid Carrier 1

Mouse models deficient in glutamate transporters have provided crucial insights into the mechanisms of RGC degeneration in NTG. In particular, mice lacking either the glutamate/aspartate transporter (GLAST) or the excitatory amino acid carrier 1 (EAAC1) spontaneously develop key features of glaucomatous neurodegeneration, including progressive RGC loss and optic nerve atrophy, despite normal IOP and anterior segment morphology, thereby closely recapitulating NTG pathology [39,40].

GLAST is predominantly expressed in Müller glial cells and plays a critical role in maintaining extracellular glutamate homeostasis in the retina [41]. Loss of GLAST function leads to impaired glutamate clearance, resulting in excitotoxic neuronal injury and increased oxidative stress due to reduced glutathione (GSH) synthesis [39,42]. Within Müller glia, glutamate is normally taken up and converted into GSH via its combination with cysteine and glycine, thus sustaining antioxidant defenses. In GLAST knockout (KO) mice, diminished glutamate uptake compromises GSH production, exacerbating oxidative damage, a key risk factor in glaucoma [43,44]. Consequently, GLAST KO mice exhibit key features of NTG due to chronic glutamate neurotoxicity and increased oxidative stress, independent of glaucoma-associated genes [45]. Phenotypically, significant RGC degeneration becomes evident in GLAST KO mice by eight months of age, with approximately 50% RGC loss. Accordingly, optic nerve cupping and thinning, together with decreased density of optic nerve axons, become manifest starting from the age of 8 months in GLAST KO mice. In parallel, the iridocorneal angle appears to be well formed, with normal Schlemm’s canal TM extent [39].

EAAC1 is mainly localized to RGCs, and its deletion causes a rapid degeneration onset detectable at 8 weeks of age, characterized by inner retina thinning and a 20% reduction in RGC density [39]. As in GLAST KO mice, the pathology in EAAC1 KO mice is attributed to a combination of glutamate neurotoxicity and oxidative stress [46]. Proteomic analyses of retinas from EAAC1-deficient mice have revealed alterations in proteins implicated in cell proliferation, metabolism, and extracellular matrix (ECM) organization, reinforcing the multifactorial nature of RGC degeneration in this context [47]. Moreover, upregulation of angiotensin II type 1 receptor and Toll-like receptor 4 in both RGCs and Müller glia suggests a contribution of the renin–angiotensin system to retinal stress responses [48].

It is important to note that in humans, although elevated vitreous glutamate levels are not consistently observed in glaucoma patients [49,50], a decline in glutamate transporter expression with age, GLAST in particular, may predispose the individual to excitotoxic damage [51,52], suggesting that the reduced function of glutamate transporters may contribute to the pathogenesis of glaucoma. Supporting the clinical relevance of these findings, rare variants in EAAT1 have been identified in POAG patients with NTG, although their functional relevance remains under investigation [53].

In a translational perspective, GLAST KO and EAAC1 KO mice have served as valuable platforms for preclinical evaluation of neuroprotective agents. In GLAST-deficient mice, compounds such as valproic acid, geranylgeranyl acetone, and astaxanthin have shown protective effects on RGCs [45,54,55]. Similarly, EAAC1-deficient mice have been found to respond to agents like candesartan, edaravone, brimonidine, ripasudil, spermidine, N-acetylcysteine, and interleukin-1 [46,48,56,57,58,59,60].

Despite certain limitations, such as diffuse rather than sectorial RGC loss and earlier onset compared to human NTG, GLAST and EAAC1 KO mice constitute important glaucoma models. They continue to offer critical insights into excitotoxicity, oxidative stress, and age-related mechanisms, contributing to RGC degeneration in glaucoma, and remain a useful platform for the development of novel neuroprotective strategies [39,61].

### 3.3. Myocilin

Mutations in the *MYOC* gene, which encodes the glycoprotein myocilin (MYOC), are a well-established cause of ocular hypertension and glaucoma, particularly POAG [62,63]. In both humans and mice, the *MYOC* gene is expressed in several ocular tissues, with the highest expression levels in TM cells. Although the exact role of MYOC is still unknown, data show that it is secreted by TM cells into the aqueous humour, where it interacts with ECM proteins, possibly participating in the maintenance of TM cell structure and function [64]. More than 90% of glaucoma-associated *MYOC* gene mutations cluster within the olfactomedin domain, a conserved structural motif essential for MYOC structure and function. Among these, the Y437H mutation has been well characterized because of its association with severe, juvenile-onset POAG in humans [65,66].

Earlier genetic models were based on the manipulation of the endogenous mouse *MYOC* gene. Notably, MYOC-null mice fail to develop ocular hypertension, RGC loss, or any other ocular abnormality [67]. Similarly, overexpression of the *MYOC* gene does not induce glaucoma-related phenotypes [68]. These findings suggested that the *MYOC* gene is not essential for the physiological regulation of IOP and that pathogenic mutations lead to a complex phenotype rather than simple toxic gain- or loss-of-function [69].

Transgenic models expressing mutated forms of MYOC have provided critical insights into the molecular mechanisms of the disease. Gould et al. [70] and Senatorov et al. [65] introduced a point mutation in the mouse *MYOC* gene corresponding to the human Y437H variant. Although the secretion of both mutant and wild-type MYOC was impaired in these mice, early and mid-life stages were not associated with significant IOP elevation or substantial RGC loss. Nonetheless, in transgenic mice expressing the mouse homolog of the Y437H mutation, glaucomatous changes became evident by 8 months of age with only mild or delayed IOP elevation. Although no pathological changes were observed in the TM, transgenic mice displayed detached cells in Schlemm’s canal adjacent to the TM and breaks in the inner wall of the Schlemm’s canal. The optic nerve of 13-month-old transgenic mice showed accumulation of darkly stained structures throughout the optic nerve, except for the peripheral areas, where patches of “empty” space without axons were observed. The loss of axonal profiles at the optic nerve periphery was concomitant with altered axoplasmic fine structure and with the presence of condensed, electron-dense axoplasm, myelin debris, or empty, swollen axonal profiles characteristic of degenerating axons in the optic nerve. The optic nerve alterations were paralleled by a 20% loss of RGCs, mainly in the peripheral retina [65]. Age-dependent pathologic alterations, including progressive decline in RGC physiological responsiveness, astrogliosis, and optic nerve degeneration, can be observed in mice expressing human MYOC^Y437H^, still independent from early IOP changes [70,71]. Interestingly, a distinct approach using adenoviral-mediated delivery of human *MYOC^Y437H^* into the iridocorneal angle results in pronounced ocular hypertension, suggesting species-specific differences, possibly linked to a peroxisomal targeting signal present in human MYOC but absent in the mouse protein [72]. Further supporting these observations, independent studies have demonstrated that the stable transgenic expression of human *MYOC^Y437H^* in mice induces IOP elevation [71,73,74]. For instance, a transgenic mouse expressing *MYOC^Y437H^* under a cytomegalovirus promoter developed reduced MYOC secretion into the aqueous humor, elevated IOP starting at 3 months of age, and subsequently resulted in RGC loss beginning at 4 months of age [73]. In addition to the Y437H mutation, other human *MYOC* variants, such as *MYOC^P370L^* and *MYOC^N450Y^*, have been characterized in knock-in transgenic mice. These models recapitulate key features of MYOC-associated glaucoma, including impaired secretion of mutant MYOC into the aqueous humor, elevated IOP, RGC loss, and visual dysfunction starting from 4 to 5 months of age [75,76]. It is noteworthy that double-mutant mice homozygous for *MYOC^Y437H^* and heterozygous for a mutated form of the antioxidant enzyme superoxide dismutase 2 exhibit earlier and more severe IOP elevation, as well as more dramatic RGC degeneration compared to mice expressing *MYOC^Y437H^* alone [77], supporting the hypothesis of an involvement of oxidative stress in the pathogenesis of MYOC-associated glaucoma. Taken together, these humanized models have provided crucial platforms for dissecting the pathogenic mechanisms underlying MYOC-associated glaucoma and serve as valuable tools for testing novel therapeutic strategies targeting mutant MYOC-induced ocular hypertension and neurodegeneration. However, these humanized models present several limitations. For instance, mice with the *MYOC^Y437H^* mutation have multiple copies of the transgene due to random integration or a mild phenotype when the transgene is inserted in the C57BL/6 mouse background. In this respect, a Cre-inducible mouse model expressing the *MYOC^Y437H^* mutation has been recently introduced. This model replicates all features of POAG, including altered TM functions followed by IOP elevation and RGC degeneration with impaired axonal transport [78], suggesting that this reliable Cre-inducible model may soon become a valuable tool for studying the pathophysiology of POAG and new therapies for its treatment.

### 3.4. Connective Tissue Growth Factor

Dysregulation of ECM homeostasis in TM is a hallmark of POAG, contributing to increased aqueous humor outflow resistance and IOP elevation. Excessive ECM deposition, together with reorganization of the actin cytoskeleton into cross-linked actin networks, promotes fibrotic stiffening of TM tissue, ultimately impairing its biomechanical properties and impeding fluid drainage [79].

Transforming growth factor-β (TGF-β), particularly the β2 isoform, plays a central role in TM remodeling, as suggested by the finding of elevated levels of TGF-β2 in the aqueous humor of glaucoma patients [80,81,82]. Once dissociated from its latent complex with latency-associated protein, TGF-β2 activates different pathways, triggering a transcriptional program promoting ECM protein production, cytoskeletal reorganization, and fibrogenic responses in TM cells [83]. One of the key downstream effectors of TGF-β2 is connective tissue growth factor (CTGF), a matricellular protein that amplifies ECM production and modulates cellular contractility [84,85]. CTGF is expressed in various ocular tissues involved in aqueous humor dynamics, including TM, ciliary body, optic nerve head, and NFL [86,87,88]. Its pathogenic role in glaucoma has been demonstrated in different in vivo models. For instance, in mice subjected to intracameral injection of adenoviral vectors expressing CTGF (AAv-CTGF), a robust IOP elevation was observed within 7 days and lasted for more than 2 months, accompanied by a significant loss of optic nerve axons. Although ECM deposition was not markedly altered, there was a pronounced increase in α-smooth muscle actin (α-SMA) expression in TM cells, indicating cytoskeletal remodeling and increased contractility [89].

A complementary genetic model further supports the role of CTGF in POAG. In transgenic βB1-CTGF mice, where CTGF is overexpressed in the lens under the βB1-crystallin promoter, elevated levels of CTGF are secreted into the aqueous humor, leading to early-onset IOP elevation starting at 1 month of age. Despite the preservation of angle structures and an anatomically open angle, these mice develop sustained ocular hypertension and progressive optic nerve axon loss. In particular, histological analyses of the optic nerves of 3-month-old βB1-CTGF mice showed areas devoid of axons, indicating axonal loss, and filled with cells of presumably glial origin. The loss of axons in optic nerves appeared to be age-dependent, indicating continuous glaucomatous damage. In addition, the histological analysis of TM revealed increased α-SMA and actin filament abundance, suggesting that CTGF induces TM stiffening through cytoskeletal modulation [89]. Beyond the outflow pathway, βB1-CTGF mice recapitulate key features of glaucomatous retinal neurodegeneration. Indeed, progressive RGC death became evident by 15 weeks of age, when the IOP increase could be measured, following axonal degeneration [90]. Increased immunoreactivity for glial fibrillary acidic protein (GFAP) and vimentin, along with elevated *Gfap* mRNA expression, indicate activation of macroglial cells, possibly reflecting early astrocytic remodeling in response to RGC injury. This neuroinflammatory environment was characterized by increased numbers of cleaved caspase-3 and TUNEL RGC-positive cells at 5–10 weeks and reduced neurofilament H staining in 10-week-old mice, indicating that RGC suffering precedes IOP increase and neuronal death [91].

Altogether, both viral and transgenic CTGF overexpression models faithfully reproduce the cardinal features of POAG, including chronic IOP elevation, TM cytoskeletal changes, optic nerve axonopathy, and RGC apoptosis. These models underscore the pathological relevance of the TGF-β2–CTGF axis in glaucomatous damage. Nevertheless, no causative mutations or polymorphisms in CTGF or related ECM-regulating genes have been identified in human POAG patients to date, suggesting a potential role for transcriptional or post-translational dysregulation rather than direct genetic causation [92].

### 3.5. Optineurin

It has been shown that about 16% of families with hereditary POAG have mutations of the *OPTN* gene on chromosome 10p14 [82,93]. In addition, 1–2% of NTG cases have been linked to *OPTN* gene mutations [82,94,95]. The *OPTN* gene codifies for optineurin (OPTN), a cytosolic protein with widespread expression and different functions, including the regulation of protein transport, the maintenance of organelle integrity, and participation in both autophagy and mitophagy [89,96]. In the eye, OPTN is expressed in multiple structures, such as cornea, TM, ciliary body epithelium, iris, and RGCs [89,97]. Different mutations in the *OPTN* gene have been identified, including E50K, H486R, and R545Q. In particular, E50K is hypothesized to be a gain-of-function mutation, and it is associated with both familial and sporadic NTG [93,98,99,100]. E50K mutation is transmitted in an autosomal dominant manner and consists of the replacement of glutamic acid at position 50 with lysine [93,100,101]. Transgenic mice have been created in order to overexpress the E50K variant under a chicken β-actin promoter combined with a cytomegalovirus enhancer. This model shows RGC death despite normal IOP levels. In particular, retinal thinning could be observed as early as 12 months of age in E50K mice. β-III tubulin-stained NFL was thinner at the optic nerve head due to loss of RGCs and their axons in the peripheral retina. Accordingly, a significant degeneration of synapses was observed in the peripheral retina, together with the loss of both amacrine and rod bipolar cells. On the other hand, both outer plexiform and outer nuclear layers (OPL and ONL, respectively) were also affected in E50K mice, with reduced expression of synaptophysin and decreased length of rhodopsin-labeled outer segments, indicating loss of rod photoreceptors and other non-RGC retinal cells, a feature that is not present in patients with POAG [102]. This peculiar characteristic has been explained by hypothesizing that the elevated expression of the E50K mutant of OPTN may lead to non-specific neurodegeneration through a toxic gain-of-function effect. To bypass this problem, a Bac transgenic mouse has been developed, in which the human E50K mutant is expressed at physiological levels. This model exhibits characteristics typical of NTG, with axonal damage, age-related loss of RGCs (but not of other retinal cell types), and impairment of visual function [103]. In particular, this model perfectly mimics patients with the E50K mutation, which is expressed in heterozygosity, carrying one mutated allele of the human *OPTN* gene and one normal allele of the mouse *OPTN* gene, making it a good model for studying the molecular mechanisms underlying POAG in general and NTG in particular and for assessing novel therapeutic interventions. However, hereditary POAG associated with *OPTN* gene mutations is a glaucoma form affecting only a minority of POAG patients; therefore, the results obtained in the E50K model cannot be generalized to the overall cohort of POAG patients.

### 3.6. Purinergic 2Y Receptors

Adenosine triphosphate (ATP) is not only essential from an energetic point of view but it is also involved in intracellular signaling pathways. In fact, ATP could bind to purinergic P2 receptors expressed by several cells, playing a crucial role in many processes including neurotransmission, muscle contraction, brain development, cell proliferation, cell death, cardiovascular regulation, and inflammation [104]. Nucleotides are released by several components of the ocular tissues, including lens, TM, retina, corneal endothelium, RGCs, astrocytes, and ciliary body [105,106,107,108,109,110,111,112]. Moreover, following IOP fluctuation, ATP is released in the eye [104], and it controls IOP level through P2 receptors [113]. P2 receptors can be divided into P2X and P2Y. There are different subtypes of P2Y receptors, among which P2Y1, P2Y2, and P2Y6 have been found to regulate IOP. These three receptors are all found in the cornea and ciliary processes. Additionally, P2Y1 is also localized in TM, P2Y2 in the retinal pigment epithelium, and P2Y6 in photoreceptors and RGCs [113,114,115,116,117]. There is evidence of elevated ATP levels in the aqueous humor of glaucoma patients, likely due to IOP elevation [118]. This suggests an involvement of dysregulated ATP signaling in IOP increase [119].

Mouse models deficient for *P2Y1* or *P2Y6* have been developed. In mice lacking *P2Y1*, there is an age-independent increase in IOP, while an age-dependent loss of RGCs can be observed. In this respect, 12-month-old P2Y1 KO mice displayed a decreased number of both Brn3a- and Rbpms-positive RGCs, with greater RGC loss in peripheral regions of the retina. These mice also showed axonal swelling and accumulated membranous organelles, as well as a significantly thinner NFL/RGC layer/inner plexiform layer. No structural abnormalities were observed by OCT in the other retinal layers, including OPL, ONL, the external limiting membrane, and the inner/outer photoreceptor segments [119]. A similar phenotype has been observed in P2Y6-deficient mice, which display IOP elevation starting from 3 months of age [120], suggesting that mice lacking *P2Y1* or *P2Y6* may be useful models for developing glaucoma treatments targeting the P2Y receptors.

### 3.7. Caveolin 1

In some patients suffering from POAG with increased IOP levels, the disease is associated with the presence of common single-nucleotide polymorphisms in *CAV1/2* genes [121,122]. These genes codify for caveolin 1 (*Cav1*) and caveolin 2, members of a family of membrane scaffold proteins, among which caveolin 1 is involved in the synthesis of caveolae [123,124]. Caveolae are invaginations of the plasma membrane rich in cholesterol and lipids and present in different cell types. In the retina, they are abundant in Schlemm’s canal and TM, as well as in the cells of the retinal vasculature and in glial cells [123,124]. These microdomains play a critical role in several cellular functions, such as signal transduction, mechanotransduction, and cytoprotection against mechanical stress [125,126,127]. Indeed, they can disassemble in response to mechanical stimulations, such as increased IOP and donate membranes for the formation of giant vacuoles to counteract the mechanical stress. This adaptive response preserves membrane integrity and prevents cellular rupture during periods of increased biomechanical load [123,124,128,129,130,131,132]. The loss of caveolin 1 in mice has been proven to compromise caveolae formation in the retina. In fact, in Cav1 KO mice in both Schlemm’s canal and TM caveolae are completely absent, and the reported reduction in aqueous humor outflow is probably responsible for the increase in IOP that can be observed starting from 5 weeks of age. In particular, IOP increase is present at 5, 12, and 24 weeks, without showing a significant increase with age. However, although the outflow pathway is dysfunctional, its morphology is largely preserved, and the anterior chamber iridocorneal angle is open [129], indicating that Cav1 KO mice may be a valuable model for studying the functional link between this risk gene and glaucoma pathophysiology.

### 3.8. Endothelin 1

Vascular endothelial cells synthesize endothelin 1 (ET-1), a potent vasoconstrictor involved in both contractility and permeability of the endothelium [133]. Both the retinal pigment epithelium and the vascular endothelium synthesize and secrete ET-1 in the retina [134]. In the eye, ET-1 dysfunction is involved in TM contraction, in the activation of astrocytes, in the altered regulation of aqueous humor outflow, and in the dysregulation of ocular perfusion [135,136,137,138,139,140,141]. Elevated levels of ET-1 have been found in the aqueous humor of patients with either hypertensive POAG or NTG [142,143,144,145], a finding that led to the hypothesis that ET-1 may contribute to glaucoma pathophysiology [146,147].

A transgenic mouse, called TET-1, which specifically overexpresses ET-1 in endothelial cells, has been developed [148]. This mouse shows an NTG-like phenotype. In fact, despite IOP levels remaining normal at all ages, at 12 months of age, the overexpression of ET-1 leads to progressive RGC death and the thinning of the NFL, inner nuclear layer (INL), and ONL, followed by the degeneration of optic nerve axons at 24 months of age. In particular, 24-month-old TET-1 mice showed a decrease in cell density in the RGC layer in both the central and the peripheral retina. RGC loss could be detected as early as 12 months exclusively in the peripheral retina, thus suggesting propagation of RGC degeneration from the peripheral towards the central retina. Concomitantly, TET-1 mice showed a progressive thinning of both the INL and the ONL, indicating severe neuronal loss not only in the RGC layer but also in the outer retina. Together with the progressive loss of RGCs, 24-month-old TET-1 mice showed NFL thinning, while degenerating axons and many vacant holes replacing the lost axons were observed in the optic nerve. Optic nerve changes were accompanied by increased GFAP expression and astrocyte disarrangement, indicative of astrocytosis in the optic nerve and indicating reactivation of glial cells following degeneration of RGC axons [149].

## 4. Models of Induced Glaucoma

As IOP elevation represents the main risk factor for glaucoma development, many models of induced glaucoma are based on IOP increase, although IOP-independent models have also been introduced to specifically study NTG. Compared to genetic models, inducible models, depending on increased IOP, allow the avoidance of possible issues related to genetic manipulation. These models often allow for modulating the extension of retinal damage and show a rapid onset of IOP elevation, thus representing useful tools for studying the pathophysiology of glaucoma and for investigating novel therapeutic approaches with timescales shorter than those required by genetic models. A wide variety of induced glaucoma models have been developed, each replicating specific aspects of the disease. The choice of the model depends on the research question and the aspect of glaucoma pathophysiology under investigation. Continuous refinement of these models is essential for the development of novel, effective glaucoma therapies. The main models of induced glaucoma are summarized in Table 3 and described below.

### 4.1. Laser Photocoagulation

One way to increase IOP, leading to the degeneration of both RGCs and optic nerves, is laser photocoagulation of TM alone or both TM and episcleral veins [150,151]. Usually, researchers use a diode laser to perform photocoagulation, while an argon laser is less used in rodent models. The main difference between the two is that the argon laser has a higher average power setting and energy density. Also, their photocoagulative effect is different due to differences in emitted wavelengths (810 nm for the diode laser and 488–514.5 nm for the argon laser). Moreover, the diode laser causes more extensive TM damage than the argon laser due to deeper and broader lesions, leading to a significantly greater rise in IOP [152]. Anterior chamber paracentesis is performed prior to laser photocoagulation. The aqueous humor is aspirated with a thin needle until the anterior chamber becomes sufficiently shallow, with peripheral iridocorneal contact. The anterior chamber’s flattening positions the TM closer to the limbal regions targeted by the laser, thereby potentially enhancing the efficacy of photocoagulation [150,153,154]. In mice, combining photocoagulation with anterior chamber flattening effectively produced the obstruction of episcleral veins, TM destruction with partial or complete obstruction of Schlemm’s canal, and development of anterior synechia. Accordingly, optic nerves displayed moderate to extensive damage of optic nerve structures, with loss and degeneration of large-diameter axons, swelling of myelin sheaths, condensation and degeneration of the cytoskeleton, and activation of glial cells in proximity to degenerating axons [151]. In Black Swiss mice, this led to a significant increase in IOP that lasted 4 to 12 weeks [150]. In C57BL/6 mice, the argon laser directed to episcleral and limbal veins resulted in an increase in IOP, which returned to baseline levels 8 weeks after laser treatment, accompanied by RGC death [155]. In the rat, laser application to limbal and episcleral veins instead of TM increased the effects of photocoagulation on the aqueous outflow blockade and ocular hypertension, which lasted for more than 1 year [156].

Although laser photocoagulation can be applied to both mice and rats, when applied to a mouse, it seems to produce more extensive retinal injuries, possibly because of the smaller size of the eye, including the thinning of both INL and ONL and photoreceptor damage. Laser photocoagulation, compared to other glaucoma models, offers a high success rate in elevating IOP within a few hours after a single treatment. RGC apoptosis is clearly measurable by 1–2 weeks and continues in a chronic pattern thereafter [150,151,152,153,154,157]. However, there are several disadvantages. Indeed, the required ophthalmic equipment is very expensive, and the operator must have highly specialized technical expertise [157]. Moreover, if multiple laser treatments are needed, corneal opacity and ocular inflammation may develop consequently [150]. Another important aspect to consider is differences in TM pigmentation, which may lead to different effects after laser photocoagulation [158]. Furthermore, a reduction in the amplitude of electroretinographic a- and b-wave has been observed, indicating that, in addition to IOP elevation, laser photocoagulation may result in unexpected effects on retinal cells different from RGCs, such as photoreceptors and/or second-order neurons [151,159]. This drawback is more evident in mice, possibly because of the smaller eye dimension with respect to the rat. Lastly, this procedure shows fluctuations and a high variability in IOP elevation magnitude, together with inconsistency in the duration of elevated pressure [150]. Nonetheless, this model induces pathological changes similar to those observed in human glaucoma, and it has been recommended to investigate neuroprotective strategies [160].

### 4.2. Episcleral Vein Cauterization

Episcleral vein cauterization (EVC) in rats is a commonly used method to induce ocular hypertension, along with other features typical of POAG. The procedure involves the cauterization of two or three episcleral vein branches to elevate IOP [161,162]. This IOP increase results from an increase in post-TM resistance caused by the occlusion of episcleral veins and is directly related to the number of cauterized veins. A hand-held cautery is used to cauterize the predetermined episcleral veins and to block their venous drainage area [163]. Usually, three veins are occluded, and this leads to RGC death, optic nerve degeneration, optic disc cupping, and thinning of NFL [161,164,165,166]. It has been observed that when only one vein is cauterized in the rat, no increase in IOP can be detected, probably because the remaining veins allow the aqueous humor to drain through alternative routes, while when four veins are cauterized, the increased IOP induces cornea decompensation and atrophy in the eye [163]. On the contrary, both the gross and the microscopic structure of the retina are preserved if two or three veins are occluded, with RGC death only observed as a consequence [164]. In particular, the increase in IOP is thought to block retrograde axonal transport, leading to RGC death through apoptosis [164,167,168]. One important aspect of this glaucoma model is that when two or three veins are occluded, there is a quick decline in the increased IOP until a plateau is reached, characterized by an ocular pressure higher than the normal pressure level, which lasts for several weeks. This probably occurs because the remaining veins and the minor collateral channels exert an accommodative effect [157]. For instance, after the EVC of three episcleral veins in the rat, IOP elevation progressively decreased starting from the 17th week after cauterization, reaching basal values at the 24th week [166]. Moreover, an initial spike of high IOP, with a 2.5-fold increase that lasted one day, followed by an IOP 1.6-fold higher than a control that lasted for 6 weeks has been registered in rats after the EVC of three veins. The same study also reported a progressive RGC loss starting 1 week after EVC and continuing at a rate of 4% RGC loss per week, more accentuated in the peripheral than in the central retina [169]. EVC has been used not only in rats but also in mice, where the increase in IOP levels lasts for 4 weeks and is followed by RGC degeneration starting 2 weeks after EVC and reaching an RGC loss of about 20% [170]. Moreover, an increase in outflow resistance is also observed, indicating that this mouse model reflects glaucoma disease progression. However, unlike rats, in mice, IOP elevation is obtained only when three veins are cauterized, while it is absent or lasts only a few days when two veins are obstructed. This difference could be explained by a more efficient compensatory mechanism in aqueous humor drainage, which restores IOP in mice faster than in rats [170]. However, the application of EVC in mice has some limitations. It is challenging from a technical point of view because of the small mouse eye globe, which makes it difficult to obtain reproducible results. In fact, the procedure may cause damage to the surrounding tissue or the sclera, thus leading to complications such as intraocular inflammation or ocular surface damage. Moreover, there is a variability in the duration of IOP elevation, which can last from 1 to 4 weeks [170]. Conversely, in rats, EVC leads to a more extended period of sustained IOP increase, which persists for at least six months [171,172]. In mice, RGC apoptosis is already present by about 2 weeks after IOP elevation, whereas in rats, RGC death is more gradual and is commonly reported at later times with measurable levels by 12 weeks after EVC [161,162,163,164,165,166,167,168,170,171,172]. In any case, IOP elevation and the amount of RGC death are similar in rats and mice, supporting the possibility of also using EVC in mice [170]. In general, EVC is a valid method for obtaining a model that recapitulates human glaucoma. In rats, RGC death is comparable to that obtained with the injection of microspheres into the anterior chamber of the eye [166]. Moreover, EVC is less invasive than laser photocoagulation and avoids anterior chamber complications [173]. Furthermore, EVC does not require intraocular manipulation or the introduction of exogenous materials, and it does not lead to ocular tissue damage or vasculature abnormalities [163,171]. In addition, this model is easily accessible and effective, and it is the model used in the majority of structural and functional studies on glaucoma [174]. However, EVC presents some disadvantages related to the cauterization procedure, such as, for instance, the risk of inducing ischemia and hyperemia [169]. Furthermore, the pathophysiology and the pattern of RGC loss differ from other models since EVC leads to a more general RGC loss, while other models mainly produce the axonal degeneration of RGCs, likely suggesting that IOP not only increases but additional factors also contribute to RGC death in the EVC model [175]. Nevertheless, the EVC rat model appears to be a reliable, cost-effective, and highly reproducible tool for investigating the pathophysiological mechanisms of glaucoma and for evaluating potential treatment strategies [169].

### 4.3. Injection of Hypertonic Saline Solutions into Episcleral Veins

This is a valuable model for simulating chronic IOP elevation [176]. Injections of hypertonic saline solution (usually 1.75 to 2 M NaCl) into the episcleral veins cause sclerosis of TM and Schlemm’s canal, resulting in altered aqueous humor outflow with subsequent IOP elevation and progressive optic nerve damage. This model is especially useful for studying the biomechanical effects of IOP elevation, axonal injury, and neurodegeneration over time, resembling human POAG. In rats, IOP elevation lower than 10 mm Hg for 2 to 34 days produced focal optic nerve lesions similar to those observed in eyes with IOP elevation ranging from 10 to 20 mmHg for less than 3 weeks. On the other hand, IOP elevations of 10–20 mmHg for more than 3 weeks, or greater than 20 mmHg for more than 1 week, often resulted in total involvement of the optic nerve. Within the optic nerve head, eyes with early damage showed axonal swelling and increased cellularity mainly at the level of the lamina cribrosa. Indications of blocked axonal transport, such as the accumulation of organelles, dense bodies, and swollen mitochondria, were also reported. While the retina was poorly affected in the presence of only partial damage of the optic nerve, the overall retinal thickness was reduced in highly affected optic nerves primarily due to loss of the entire RGC layer and NFL. The anterior segment of the eyes with elevated IOP showed anterior synechiae; loss of the normal TM architecture; and patency of Schlemm’s canal, collector channels, and veins of the limbal vascular plexus [160].

In injected animals, IOP elevation typically begins within 3–7 days after injection and may persist for several weeks to months [160,176,177]. Rats with prolonged high IOP showed diminished amplitudes of electroretinogram components, suggesting damage to bipolar and photoreceptor cells, similarly to those observed in the late phase of chronic glaucoma patients. In addition, these rats showed alterations in the positive scotopic threshold response, indicating RGC dysfunction [178]. Studies have shown that elevated IOP leads to significant RGC death over several weeks following IOP elevation [179]. This model of induced glaucoma has been extensively used for assessing the efficacy of potential neuroprotective therapies on chronic glaucomatous disease [180]. For instance, it has been used to demonstrate the effectiveness of nerve growth factor eye drops in reducing RGC apoptosis [179]. However, this model is technically complex, requiring microsurgical skills to cannulate episcleral veins. Therefore, it is primarily used in rats due to their larger episcleral veins compared to mice. Moreover, a certain variability in IOP response was observed among subjects since not all treated eyes develop a significant IOP increase, and variability exists in the magnitude and duration of IOP elevation depending on injection precision and animal responses [176].

### 4.4. Injection of Microbeads or Viscous Materials into the Anterior Chamber

The microbead occlusion model is a widely utilized method to induce IOP-dependent chronic ocular hypertension [181,182,183]. Injection of inert microbeads (typically polystyrene or magnetic, 10–15 µm in diameter) into the anterior chamber creates a physical blockade of aqueous outflow pathways, with subsequent obstruction of TM and Schlemm’s canal. This barrier impedes aqueous humor drainage, leading to sustained IOP elevation and RGC degeneration. Variations in bead size and concentration allow the fine-tuning of IOP levels and durations that may extend 2–4 weeks after a single injection up to 8 weeks after two injections. However, the model requires repeated injections to maintain elevated pressure over time. Studies performed in Sprague Dawley rats reported that after latex microspheres injection into the eye anterior chamber, IOP was elevated approximately 30% above baseline, persisting for several weeks [166]. The mean IOP increases, and the duration of ocular hypertension could be enhanced by the concomitant injection of microbeads with a viscoelastic material such as sodium hyaluronate or hydroxypropylmethylcellulose [166,182,184]. A further improvement of this technique is the use of magnetic microbeads that can be guided by a magnet to optimize the blockade of TM [185,186]. Injecting microbeads into the anterior chamber of the rodent eye led to RGC apoptotic death within 3 weeks of the injection [182,184]. This type of model has been applied primarily to rats because of the technical difficulty of manipulating the significantly smaller mouse eye [166]. TM occlusion through the use of microbeads has been used to test neuroprotective treatments on glaucoma [187]. This induced glaucoma method has the advantage of producing low levels of ocular inflammation, hence preserving the functional integrity of the eye. Otherwise, it requires precise injection techniques to ensure consistent bead placement and IOP elevation. Mice receiving anterior chamber injections of 10 μm microbeads displayed IOP elevation over time, together with a drop in axon density and a significant reduction in RGC number and density. The RGC loss in this model appeared clustered and especially prominent in the peripheral retina on a background of diffuse loss [188].

The physical occlusion of aqueous outflow pathways can also be obtained by injecting viscous materials into the anterior chamber. For instance, a single injection of hyaluronic acid in the rat eye induced an increase in IOP that lasted 8 days, while injecting hyaluronic acid once a week for 9 weeks resulted in a sustained increase in IOP that reached a plateau on the 4th week after the first injection [189]. An additional viscoelastic material used to induce ocular hypertension is methylcellulose. Previously developed in rabbits [190], this method has been successfully applied to both rats [191,192,193] and mice [194,195,196]. The model is obtained by injecting the anterior chamber with 2% methylcellulose. After the injection, there is a rise in IOP that stabilizes within 24 h, and IOP elevation is maintained for up to 2 weeks [194], when RGC death can be evidenced [192,193,194,195,196]. This model is useful for studying human glaucomatous neurodegeneration and testing the effectiveness of treatments to reduce or prevent RGC neurodegeneration [191,192,193,194,195,196].

### 4.5. Intravitreal Injections of Excitotoxic Substances

Intravitreal injections of various agents directly into the vitreous humor of the eye lead to RGC loss and optic nerve damage in an IOP-independent fashion, thus modeling NTG, where IOP remains within the normal range [197]. In these models, intravitreally injected glutamate, glutamate analogs such as N-methyl-D-aspartate (NMDA), or kainate induce excitotoxicity, overstimulating glutamate receptors and leading to increased calcium influx, oxidative stress, and RGC apoptosis in a dose-dependent manner [177,198]. Acute models, obtained with a single injection of high-dose NMDA (150–200 nmol), show RGC apoptosis 1 h after the injection [199,200], while chronic models, obtained with injections of low-dose NMDA (2–20 nmol), lead to minimal RGC damage, which can be increased by repeating the low-dose treatment [199,201,202]. These graded responses allow the adjustment of the severity of injuries to specific experimental needs [203]. In rats, intravitreal injections of high-dose NMDA triggered a reduction in the RGC-specific marker Thy-1 and the depletion of more than 80% of RGCs [204,205,206,207,208,209]. On the other hand, NMDA-induced toxicity not only affected rat RGC survival but also triggered neuronal apoptosis in the INL and caused a reduction in both scotopic and photopic electroretinograms, suggesting that, besides inducing RGC degeneration, excitotoxic agents also act on post-photoreceptor neurons [210,211].

Although the contribution of excitotoxicity in the development of human glaucoma is still a matter of debate due to conflicting results on glutamate levels in the vitreous of glaucoma patients [49,50], the method of intravitreal injections of excitotoxic substances offers some advantages over other methods, as it produces damage localized to the retina generally without affecting other ocular structures. Moreover, it allows a controlled onset, enabling the precise timing of RGC degeneration [212]. However, this method shows a wide variability of success, both interindividual and interspecies, since it is known that the administration of the drug through this technique is influenced by anatomical differences of the eyes and by the injection site and may induce damage to cells different from RGCs [210,213]. In addition, these models mimic acute glaucoma and do not replicate the chronic progression of human glaucoma [198]. Nevertheless, these models have provided useful insights into the effectiveness of neuroprotective interventions to reduce excitotoxicity-induced damage of RGCs [214].

### 4.6. Axonal Injuries

Axonal injury models simulate glaucomatous optic neuropathy by inducing direct mechanical damage to optic nerve axons, leading to RGC loss and mimicking key features of glaucoma-related neurodegeneration [215]. Following axonal injuries, RGC apoptosis typically progresses through two phases of cellular degeneration. During primary degeneration, RGCs are damaged by oxidative stress within a few hours after injury, suggesting an acute cellular response. During secondary degeneration, progressive RGC apoptosis occurs within 3 to 6 months [215,216,217,218]. The most common axonal injury models are made by crush or transection of the optic nerve [177]. In the first one, the optic nerve is mechanically crushed with calibrated forceps, thus causing acute injury and retrograde degeneration of RGCs. It is generally used to study mechanisms of axonal degeneration and to test neuroprotective or regenerative therapies. In the second one, the optic nerve is completely cut, leading to rapid loss of RGC axons. This model is useful for studying Wallerian degeneration and mechanisms of neuronal death without confounding factors such as elevated IOP. A further model of axonal injury is that of partial optic nerve transection, in which only a part of the optic nerve is transected to study the effects of an incomplete axonal injury. In particular, this model allows the investigation of secondary RGC degeneration that in humans can be observed after IOP increase, which is responsible for primary RGC degeneration [217].

Although the axonal injury models lack the progressive nature of human glaucoma, they are suitable for the analysis of neurodegenerative processes that act independently of IOP changes, providing insight into the effects of axonal transport interruption accompanied by glial cell activation and neuroinflammation [218].

### 4.7. Other Experimental Models

Several alternative models have been developed to induce glaucoma-like changes.

One of these models is based on chronic exposure to glucocorticoids, known to induce glaucoma in a significant number of patients, which can cause secondary glaucoma by altering TM function [219,220]. In rodents, three models, different in the modality of drug administration, have been reported. In the topical model, glucocorticoid drops are applied to the eyes for 6 weeks. Although the amount of IOP elevation shows some variability, this model develops evident glaucomatous optic nerve changes. These changes are less detectable in a second model of glucocorticoid administration, where the drug is systemically delivered, likely because IOP elevation is lower than that measured in the topical model [177,221,222,223]. In the third model, animals are exposed to periocular steroid treatment. Through this administration route, changes in outflow capability have been observed in enucleated eyes [224]. In rodents, IOP elevation occurs earlier than in larger animals, usually within 1–3 weeks after glucocorticoid exposure. In mice, RGC loss can be observed 2–4 weeks after IOP elevation, with progressive degeneration continuing in the following weeks [223,224].

The circumlimbal suture model is a model in which the mechanical obstruction of aqueous outflow is obtained through a microsurgical suture applied from the conjunctiva to the limbus, ultimately resulting in sustained IOP elevation [225,226]. Circumlimbal suture insertion in mouse eyes resulted in a chronic IOP elevation in the absence of angle closure, which correlates with increased episcleral venous pressure [226]. This is a method that ensures an increase in IOP lasting at least 8 weeks in rats and 12 weeks in mice [225]. In mice, apoptotic RGCs appear approximately 1–2 weeks after IOP elevation, while cell loss increases in the following weeks [225,226].

The experimental autoimmune glaucoma is an immune-based model induced by systemic immunization with ocular antigens (e.g., heat shock proteins). This model reflects the autoimmune component hypothesized in glaucoma pathogenesis, characterized by microglial activation and antibody deposition in ocular tissues [227,228]. For instance, immunization with an optic nerve homogenate in the rat leads to gliosis and apoptotic RGC degeneration in the absence of IOP elevation [229]. In mice, RGC apoptosis is detectable within the first week after treatment, progressing over weeks [228,229].

In the ischemia–reperfusion (I/R) injury model, a transient IOP elevation (e.g., to 120/140 mmHg for 60 min) is obtained in rodents by the cannulation of the anterior chamber of the eye to induce ischemia followed by reperfusion. This model is characterized by extremely high IOP, intense oxidative stress, and RGC degeneration, which simulate acute glaucomatous events, although degeneration can also be observed in retinal cells different from RGCs [230,231]. RGC apoptotic degeneration can be observed within 24 h after ischemia induction, with a progression up to 7 days [227,228], indicating that RGC death in the I/R model occurs early and continues over time.

## 5. Discussion

Glaucoma is a significant threat to human vision. It is a complex and multifactorial disease with genetic and environmental components, for which its weight can hardly be defined. In addition, the mechanisms underlying glaucoma pathogenesis remain not fully understood. Moreover, from a therapeutic point of view, current treatments are focused on the only modifiable risk factor, IOP, and IOP-lowering drugs are used not only to treat glaucoma patients with overt IOP elevation but also those suffering from NTG. Therefore, current treatments may be insufficient for preventing disease progression, which in some glaucoma patients may proceed to irreversible blindness despite the use of IOP-lowering drugs. The time window between the beginning of RGC suffering and RGC death may be used to treat glaucoma patients with interventions to promote neuroprotection and neuroenhancement and, hopefully, to decrease neurodegeneration. A better comprehension of glaucoma pathophysiology, including the elucidation of the signaling pathways involved in RGC death and optic nerve damage, and the development of new treatments, not only aimed at better IOP control but also at stimulating optic nerve protection and regeneration, are urgently needed. In this respect, the use of rodent models of glaucoma has provided paramount support to our knowledge of the disease. Mice carrying genetic mutations leading to spontaneous IOP increase and glaucoma development have been particularly useful for studying the effect of chronic IOP elevation on RGCs and optic nerve, while models of experimentally induced glaucoma have allowed the study of the effect of acute injuries on RGCs over a short period of time. Several models yield similar phenotypes, as expected for a multifactorial disease such as glaucoma. In this respect, some features (for instance, the mimicking of a particular type of human glaucoma such as POAG) are typical of genetic models, while others (for instance, the progressive loss of RGC and optic nerve damage) are spread between genetic and inducible models. Table 4 summarizes the main features of the different rodent models of glaucoma described in this review, highlighting differences and similarities between the various models.

**Figure 2:**
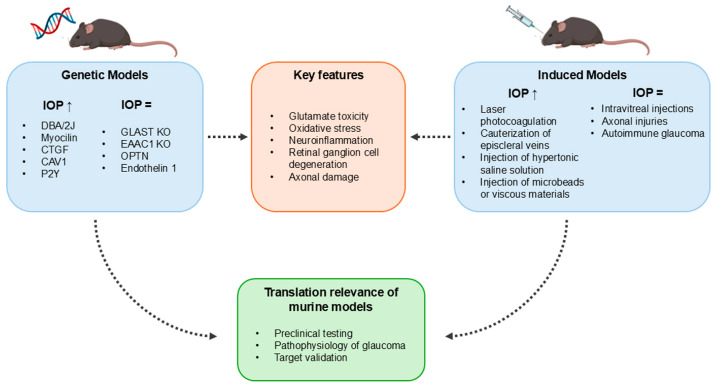
Key features and translational relevance of genetic and induced models of glaucoma.

It is clear that no single model may represent human glaucoma, as each model has its own particular features that only allow for responding to specific experimental questions. However, although each single model may present advantages and disadvantages, rodent models as a whole may provide valuable information about the molecular mechanisms involved in the development and progression of different glaucoma forms, as well as on the effectiveness of new therapeutic approaches (Figure 2). In this respect, basic studies need to be expanded to enable easier translation to clinical studies, and new rodent models focused on specific aspects of the disease may help identify cause-and-effect relationships between molecular alterations and glaucomatous changes in the retina.

## 6. Conclusions

The exact molecular mechanisms leading to glaucoma are still elusive, and rodent models of the disease, although of primary importance for investigational research, fail to provide a comprehensive answer on how the disease develops and progresses. Therefore, the development of more complex rodent models may be helpful for pre-clinical studies. Valuable hints for the development of advanced models of human-like glaucoma may be derived from the study of human susceptibility to the disease. For instance, as recently reviewed by Anwar and colleagues [232], a clear association exists between glaucoma susceptibility and lineage. Indeed, African descents display a higher susceptibility to the disease due to central corneal thickness, corneal hysteresis, and variations in the properties of optic discs. This overt difference has led to the identification of genetic determinants, relative to which knowledge has been significantly expanded with tools like genome-wide association studies (GWASs). Using GWAS, 329 genetic loci have been associated with POAG, highlighting the major contribution of common genetic variants to this disease. On the other hand, the same common variants typically have small effect sizes, thereby remaining poorly exhaustive per se in explaining the disease’s physiopathology and heritage. Furthermore, variants and genes highlighted by GWAS might not have a direct biological relevance to the disease, thus raising the need for functional validation to establish the interplay between variant genes and molecular mechanisms in glaucoma pathogenesis [233]. Therefore, advances in the study of genetic determinants of glaucoma open the possibility of generating novel and more precise experimental models both in vivo and in vitro. On the other hand, additional efforts are still required to effectively unravel genes and regulatory networks in order to be implemented in a possibly effective self-standing in vivo model of glaucoma.

## Figures and Tables

**Table 1:** Medications used for the treatment of glaucoma.

Class	Medications	Mechanism of Action
α2-adrenergic agonists	Brimonidine Apraclonidine	Reduction in aqueous humor production Increase in aqueous humor outflow
β-adrenergic antagonists	Timolol Carteolol Levobunolol Metipranolol Betaxolol	Reduction in aqueous humor production
Cholinergic agonists	Pilocarpine Carbachol	Increase in aqueous humor outflow
Carbonic anhydrase inhibitors	Dorzolamide Brinzolamide Acetazolamide Methazolamide Dichlorphenamide	Reduction in aqueous humor production
Prostaglandin analogs	Latanoprost Travoprost Bimatoprost Tafluprost Unoprostone Latanoprostene bunod	Increase in aqueous humor outflow
Rho kinase inhibitors	Netarsudil	Reduction in aqueous humor production Increase in aqueous humor outflow Reduction in episcleral venous pression
Hyperosmotic agents	Glycerol Mannitol Isosorbide	Reduction in aqueous humor volume by moving water out of the eye and into the blood (these medications are used for the short-term management of acute glaucoma)

**Table 2:** Main genetic models of glaucoma and their features.

Model	Glaucoma Phenotype	IOP	RGC Loss	Structural Alterations	Onset	Notes
DBA/2J	Pigment dispersion glaucoma	High	Progressive and severe	Pigment dispersion, angle closure, optic nerve atrophy	6–9 months	Widely used; spontaneous degeneration; inter-individual variability
GLAST KO	NTG	Normal	Significant and early	RGC degeneration, oxidative stress, glial activation	~3 months	Mimics retinal damage from oxidative stress and glial dysfunction
EAAC1 KO	NTG	Normal	Early and progressive	Early RGC loss, oxidative damage	~3 months	Glutamate excitoxicity model; no IOP elevation
MYOC Y437H	Juvenile-onset POAG	High	Axonal degeneration	TM dysfunction, ER stress, intracellular myocilin aggregates	2–4 months (variable)	Humanized knock-in mutation; relevant for genetic studies
βB1-CTGF	POAG	High	Axonopathy and RGC apoptosis	α-SMA increase, altered TM cytoskeleton, glial activation	1 month	Lens-specific CTGF secretion; ECM and TM remodeling
AAv-CTGF	POAG	High from day 7 post-injection	Axonal loss by 2 months	α-SMA increase in TM, cytoskeletal reorganization, altered TM ultrastructure	Days to weeks after injection	Acute, inducible model
OPTN	NTG	Normal	Progressive	Axonal damage, retinal thinning	4–6 months	Mimics *OPTN* mutation-associated NTG
P2Y	POAG	High	Progressive	Axonal degeneration, retinal thinning	Variable	Highlights role of purinergic signaling
Cav1	POAG	High	Progressive	Loss of caveolae in Schlemm’s canal/TM, reduced aqueous outflow	Variable	Biomechanical dysfunction
ET-1	NTG	Normal	Progressive	Vascular changes, optic nerve degeneration; retinal thinning	~12 months	Vascular contribution to NTG

IOP, Intraocular pressure; RGC, retinal ganglion cell; GLAST, glutamate/aspartate transporter; KO, knockout; NTG, normal tension glaucoma; EAAC1, excitatory amino acid carrier 1; MYOC, myocilin; POAG, primary open-angle glaucoma; TM, trabecular meshwork; ER, endoplasmic reticulum; CTGF, connective tissue growth factor; SMA, smooth muscle actin; ECM, extracellular membrane; OPTN, optineurin; P2Y, purinergic receptor 2Y; Cav1, caveolin 1; ET-1, endothelin 1.

**Table 3:** Main models of induced glaucoma and their features.

Model	Glaucoma Phenotype	IOP	RGC Loss	Structural Alterations	Onset	Notes
Laser photocoagulation	IOP-dependent	High	Progressive	TM damage; Optic nerve degeneration; inflammation	Rapid onset (days)	Mimics human high-tension glaucoma; requires specialized technique
Episcleral vein cauterization	IOP-dependent	High	Variable, comparable between rats/mice	Outflow resistance increase; axonal and optic nerve damage	Days to weeks	Reproducible; technically challenging in mice
Hypertonic saline injection	IOP-dependent	High	Progressive	TM and SM sclerosis	Weeks to months	Valuable for biomechanical and chronic IOP studies; technically demanding in mice
Microbead/viscous material injection	IOP-dependent	High	RGC loss (~25–38%)	Physical TM blockage	Days to weeks	Adjustable IOP elevation; low inflammation; repeated injections may be needed
Intravitreal excitotoxins	IOP-independent	Normal	Acute, dose-dependent	Direct RGC injury via excitotoxicity	Immediate	NTG model; may also affect retinal neurons other than RGC
Axonal injury (crush/transection)	IOP-independent	Normal	Rapid, severe	Direct axonal damage; Wallerian degeneration	Immediate	Useful for neurodegeneration and regeneration studies
Steroid-induced methods	IOP-dependent	Moderately elevated	Gradual, variable	TM function alteration; optic nerve degeneration	Weeks	Different modality of drug administration; high variability
Circumlimbal suture	IOP-dependent	High	Progressive	Outflow resistance increase; optic nerve degeneration	Weeks to months	Sustained IOP elevation
Experimental autoimmune glaucoma	IOP-independent	Normal	Progressive	Immune-mediated RGC degeneration; microglial activation	Days to weeks	Mimics autoimmune glaucoma pathogenesis
Ischemia–reperfusion injury	IOP-dependent	High but transient	Rapid	Retinal cell degeneration	Days	Acute model of ischemic damage; not chronic glaucoma

**Table 4:** Similarities and differences in the main features of rodent models of glaucoma.

IOP Levels	Model	Type of Human Glaucoma Mimicked	RGC Damage	Onset
High	AAv-CTGF	POAG	Progressive	Days to weeks
	BB1-CTGF			1 month
	MYOC Y437H			2–4 months
	P2Y			4–6 months
	Cav1			variable
	Laser photocoagulation	Secondary		Days
	EVC			Days to weeks
	Microbead/viscous material injection			
	Hypertonic saline injection			Weeks to months
	Circumlimbal suture			
	DBA2J			6–9 months
High but transient	I/R injury		Rapid	Days
Moderately high	Steroid-induced methods		Progressive	Weeks
Normal	Intravitreal cytotoxin	NTG	Acute	Immediate
	Experimental autoimmune glaucoma		Progressive	Days to weeks
	Axonal injury			Weeks to months
	GLAST KO			About 3 months
	EAAC1 KO			About 3 months
	OPTN			4–6 months
	ET-1			About 12 months

EVC, Episcleral vein cauterization; I/R, ischemia/reperfusion.

## Data Availability

Not applicable.

## Abbreviations

The following abbreviations are used in this manuscript:

RGCs	Retinal ganglion cells;
NFL	Nerve fiber layer;
IOP	Intraocular pressure;
TM	Trabecular meshwork;
NTG	Normal tension glaucoma;
POAG	Primary open-angle glaucoma;
PACG	Primary angle-closure glaucoma;
GLAST	Glutamate/aspartate transporter;
EAAC1	Excitatory amino acid carrier 1;
GSH	Glutathione;
KO	Knockout;
ECM	Extracellular matrix;
TGF-β	Transforming growth factor β;
CTGF	Connective tissue growth factor;
α-SMA	α smooth muscle actin;
GFAP	Glial fibrillary acidic protein;
OPL	Outer plexiform layer;
ONL	Outer nuclear layer;
P2Y	Purinergic 2Y receptors;
ATP	Adenosine triphosphate;
Cav1	Caveolin 1;
ET-1	Endothelin 1;
INL	Inner nuclear layer;
EVC	Episcleral vein cauterization;
NMDA	N-methyl-D-aspartate;
I/R	Ischemia/reperfusion;
GWAS	Genome-wide association studies.
